# Impact evaluation of a healthy lifestyle intervention to reduce cardiovascular disease risk in health centers in San José, Costa Rica and Chiapas, Mexico

**DOI:** 10.1186/s12913-015-1248-7

**Published:** 2015-12-28

**Authors:** Meredith P. Fort, Sandra Murillo, Erika López, Ana Laura Dengo, Nadia Alvarado-Molina, Indira de Beausset, Maricruz Castro, Liz Peña, Manuel Ramírez-Zea, Homero Martínez

**Affiliations:** Department of Family Medicine, University of Colorado, 13055 East 17th Avenue, Aurora, CO 80045 USA; INCAP Research Center for the Prevention of Chronic Diseases, Guatemala City, Guatemala; School of Nutrition, University of Sciences and Arts of Chiapas, Tuxtla Gutiérrez, Chiapas Mexico; School of Nutrition, University of Costa Rica, San José, Costa Rica; RAND Corporation, 1776 Main Street, Santa Monica, CA USA; Hospital Infantil de México “Dr. Federico Gómez”, Mexico City, Mexico

**Keywords:** Cardiovascular disease, Health promotion, Primary care, Hypertension, Type 2 diabetes, Mesoamerica

## Abstract

**Background:**

Previous healthy lifestyle interventions based on the *Salud para Su Corazón* curriculum for Latinos in the United States, and a pilot study in Guatemala, demonstrated improvements in patient knowledge, behavior, and clinical outcomes for adults with hypertension. This article describes the implementation of a healthy lifestyle group education intervention at the primary care health center level in the capital cities of Costa Rica and Chiapas, Mexico for patients with hypertension and/or type 2 diabetes and presents impact evaluation results.

**Methods:**

Six group education sessions were offered to participants at intervention health centers from November 2011 to December 2012 and participants were followed up for 8 months. The study used a prospective, longitudinal, nonequivalent pretest-posttest comparison group design, and was conducted in parallel in the two countries. Cognitive and behavioral outcome measures were knowledge, self-efficacy, stage-of-change, dietary behavior and physical activity. Clinical outcomes were: body mass index, systolic and diastolic blood pressure, and fasting blood glucose. Group by time differences were assessed using generalized estimating equation models, and a dose–response analysis was conducted for the intervention group.

**Results:**

The average number of group education sessions attended in Chiapas was 4 (SD: 2.2) and in Costa Rica, 1.8 (SD: 2.0). In both settings, participation in the study declined by 8-month follow-up. In Costa Rica, intervention group participants showed significant improvements in systolic and diastolic blood pressure and borderline significant improvement for fasting glucose, and significant improvement in the stages-of-change measure vs. the comparison group. In Chiapas, the intervention group showed significant improvement in the stages-of-change measure in relation to the comparison group. Significant improvements were not observed for knowledge, self-efficacy, dietary behavior or physical activity. In Chiapas only, a significant dose–response relationship was observed for systolic and diastolic blood pressure.

**Conclusion:**

Group education interventions at health centers have the potential to improve stage-of-change activation, and may also improve clinical outcomes. In the future, it will be essential to dedicate resources to understand ways to reach a representative group of the patient population, tailor the intervention so that patients are engaged to participate, and consider the broader family and community context that influences patients’ capacity to manage their condition.

## Background

Cardiovascular diseases are the leading cause of death globally, and place an increasing burden on low and middle-income countries [1–3]. Arterial hypertension and type 2 diabetes, the focus of this study, are principal risk factors for cardiovascular disease (CVD), and represent a major cost to health care systems in the Mesoamerican region [4, 5]. According to the Costa Rican Social Security Institute, the estimated disease prevalence in Costa Rican adults for diabetes is 10 % and for hypertension is 31.5 % [6]. Based on estimates from the 2012 Mexican National Health and Nutrition Survey, 9.2 % of adults have diabetes, and 31.5 % have hypertension [7].

In 1994 the National Heart, Lung and Blood Institute (NHLBI) of the US National Institutes of Health launched an initiative called “Health for Your Heart” (*Salud Para Su Corazón* in Spanish) to provide a federal response to CVD risk being the number one cause of death among Hispanics [8, 9]. One part of the initiative was a heart-healthy curriculum designed to train community health workers to teach community residents about preventing risk factors and adopting heart healthy behaviors, which was developed and evaluated in Hispanic populations in the US. Key aspects of heart healthy behavior are captured in a publication by the US Health and Human Services titled “Your Guide to a Healthy Heart” [10].

From 2007–2009 NHLBI funded a pilot intervention study in an urban community health center in Guatemala based on an adaptation of *Salud Para Su Corazón*. This experience demonstrated that implementation was feasible in a Guatemalan public primary care health center, and resulted in significant reductions in systolic and diastolic blood pressure, and significant improvements in knowledge and behavior related to reducing cardiovascular disease risk [11]. In 2009, with funding from NHLBI’s Office of Global Health and the United Health Group, the Institute of Nutrition of Central America and Panamá (INCAP) in Guatemala launched CIIPEC, a regional Center of Excellence focused on the prevention of chronic diseases in Mesoamerica [12].

Based on the pilot intervention study in Guatemala, CIIPEC worked with the Schools of Nutrition of the University of Costa Rica and the University of Sciences and Arts of Chiapas, the Costa Rican Social Security Institute, and the Mexican Secretary of Health to adapt and implement the cardiovascular risk reduction intervention model to urban primary care health centers in San José, Costa Rica and Tuxtla Gutiérrez, Chiapas, Mexico from 2011–2012. This article describes the adapted intervention and presents impact evaluation results.

## Methods

### Description of the Intervention

The title of the study was “Primary health and community-based support model to lower the risk of cardiovascular diseases in individuals with type 2 diabetes mellitus and/or arterial hypertension in urban areas of San José, Costa Rica and Tuxtla Gutierrez, Chiapas”.

Starting in 2011, researchers based at the Schools of Nutrition of the University of Costa Rica and the University of Sciences and Arts of Chiapas conducted a formative research phase including focus group discussions and key informant interviews to gain an understanding of patient knowledge and perceptions of chronic disease and the health care service delivery context in each of the two settings [13]. Jointly, the research teams in both settings defined the content for the educational sessions, drawing on the CVD risk reduction education materials from the previous study conducted in Guatemala; content that was considered to be most relevant and important, based on focus group discussions with patients and interviews with health care providers, was organized into six lessons. The method for teaching was also defined jointly and described in a manual for facilitators. Then, each research team adapted the training materials to the local context, based on what was learned during the focus group discussions. During the adaptation process, appropriate terms and names that are typically used in each setting were changed in the education materials. The materials were then validated to ensure that they would be understood. The result of this process were two sets of manuals and teaching materials, with the same content and lesson plans, but with adaptations appropriate for each setting. A project name and logo was developed in each site: *Corazón Pura Vida* in San José, Costa Rica and *Corazón* and *Sano y Fuerte* in Chiapas, Mexico.

Following the adaptation and validation process, the research team conducted training sessions with health care staff based at the intervention government primary care health centers in each of the two settings. In each setting, two health centers were selected for the intervention. Health promoters (in both countries, paid employees within the formal health care system) and other health center personnel that work with patients with chronic conditions (nurses, nutritionists, and physicians) participated in training sessions with the study team -- faculty from the School of Nutrition in each setting. Training sessions for health center staff were conducted in the primary care health centers and were each 2 hours long.

The training sessions focused on the use of the adapted manual and educational materials and key aspects of conducting group education sessions; the aim was to prepare health care staff both to share content to increase patient knowledge and also to promote patient-level behavior change. Training for the health care workers began with two sessions focused on the facilitation of participatory, situated group education sessions and the format to be followed as outlined in the manual. Situated learning takes into account and is situated in people’s context, their cultural understanding of health and disease, their concerns, vocabulary, resources, and their daily experience [14]. Then prior to each of the six group education sessions with patients, a training session for health care staff was offered on the specific content of that session, where the research team modeled the educational session, followed by active discussion. Halfway through the intervention, the research team offered a feedback session, to give the health care staff an opportunity to reflect and reorient their work so that they could better manage the group sessions according to situated education. The extent to which the healthcare workers adhered to the protocol was noted by an observer from the research team and quality criteria were used to assess each of the sessions; an explanation of these criteria and the process that was used will be reported in a forthcoming publication.

Health promoters, supported by other health care personnel from their primary care team, were responsible for organizing and conducting the 2-hour group education sessions with patients about CVD, risk factors, and healthy heart behavior. The sessions were designed to be interactive and were no larger than 20 patients. Patients were able to participate during any of the educational sessions once they were enrolled in the study, and did not have to follow the same sequence of educational sessions, in order to incorporate new patients. All participants had the opportunity and were encouraged to participate in six educational sessions led by health promoters and other members of the primary care team, and during which one or more members of the research team was also present. Each one of the six sessions was offered multiple times in order to allow participants to have the opportunity to take part if they missed one of the sessions. The summary box (Table 1) presents the six sessions, content, and the aim of the session and expected changes in participants.

**Table 1:** Summary Box: Healthy Lifestyle Education Sessions

Session Title	Content	Aim of the session
I want my heart to be healthy and strong	•Modifiable and non-modifiable cardiovascular risk factors and protective factors •Stages of change and how to overcome barriers	Participants will be able to relate lifestyle habits with risk factors and protective factors for chronic disease development, and be prepared to make changes.
Healthy eating with the family	•Healthy diet •Food groups •Quantity, quality and variety	Participants will develop a conceptual understanding of healthy diet and how to apply it at home.
I manage my blood pressure	•What is blood pressure and how it affects the body and its impact on work, social life and the family •Normal blood pressure •Recommendations and dietary changes •Reading food labels	Have participants feel that they are able to improve their diet to control their blood pressure.
I manage my diabetes	•What is diabetes •Carbohydrates and glycaemia •Easy steps for a diet for people with diabetes •Medications for diabetics and treatment recommendations	Participants will understand basic concepts about diabetes, and self-management focusing on diet and medications.
Steps for having a healthy and strong heart	•What are cholesterol and triglycerides •Normal and elevated values •Recommendations for lifestyle change •Dietary change and recipes	Participants will be able to identify resources within reach to incorporate self-care and lifestyle change into their lives.
Say yes to physical activity and self-care	•Physical activity •Mental health •Communication with the family	Participants will be able to identify healthy behavior for maintaining a healthy heart and preventing complications.

Source: Manual for *Corazón Pura Vida* (San José, Costa Rica) and *Corazón Sano y Fuerte* (Chiapas, Mexico), 2011

At baseline, all participants were classified as having low, medium, or high global cardiovascular disease (CVD) risk, using a World Health Organization risk prediction chart [15]. The WHO risk prediction chart uses easy-to-measure parameters: sex, age, blood pressure, presence or absence of diabetes, and tobacco use. The chart indicates total 10-year risk of a fatal or non-fatal cardiovascular event (myocardial infarction or stroke) and according to the 10-year risk of a CVD event, patients are stratified as follows: low risk is <10 %; medium risk is 10 % to < 20 %; and high risk is 20 % to < 30 % and very high risk (coupled with high risk in this case) is 30 %.

To reinforce what patients were learning in the health education sessions, home visits were made by the research team to patients with medium or high risk. In Costa Rica, high-risk patients did not receive a home visit because as a part of the usual care practice they were expected to show up for check-ups at the health center more frequently than medium and low risk patients. In both countries, all intervention participants were called on a monthly basis to remind them to participate in the educational sessions.

**Fig. 1:**
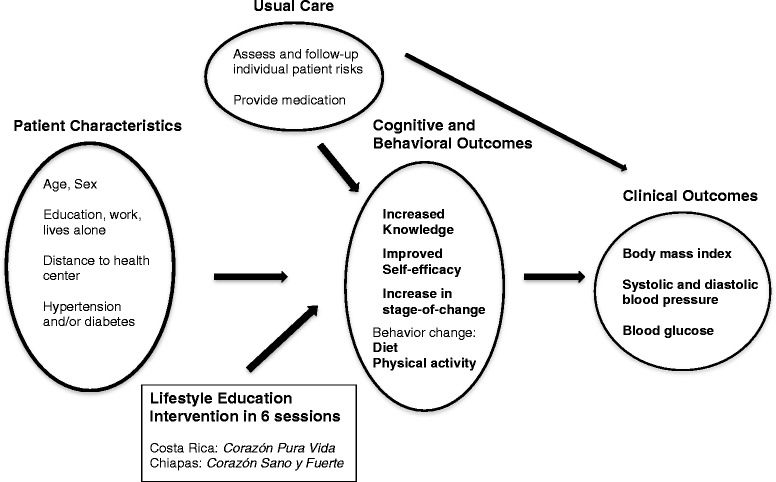
Conceptual model for the lifestyle education intervention in San José, Costa Rica and Chiapas, Mexico

Figure 1 presents the conceptual model for the healthy lifestyle intervention to reduce the risk of cardiovascular disease implemented in government primary care health centers in San José, Costa Rica and Tuxtla Gutiérrez, Chiapas, Mexico. The intervention draws on three theoretical frameworks: the Health Belief Model [16], Social Learning theory [17] and the Trans-theoretical model [18]. The conceptual model for the intervention posits that participants exposed to the group education sessions would be more likely to have increased knowledge, improved self-efficacy, and be in a higher stage-of-change (in contrast to patients in the comparison group) and these changes in cognitive factors could lead to increased healthy behavior, and in turn, clinical outcomes might improve. The analytic framework and empirical analyses do not measure exactly all of the steps in the model; other techniques including path analyses would have been required. Individual patient characteristics (age, sex, years of formal schooling, distance to the health center, working, living alone, and diabetic and/or hypertensive disease status) may influence knowledge, self-efficacy, stage-of-change and behavior, and are therefore included in the model as potential confounders to be controlled for in the analysis [19].

### Study design

The study used a prospective, longitudinal, nonequivalent pretest-posttest comparison group design, and was conducted in parallel in each of the two countries. The health education intervention for patients was implemented from November 2011 to December 2012. Participants were enrolled in the study during a 4-month period and were followed-up over a period of 8 months with data collected at baseline, mid-point, and 8 months. The 8-month follow up was determined as the shortest time frame that would allow patients to participate in the 6 different educational sessions. Data were also gathered for participants in a usual care group at the same time points as for the intervention group.

### Setting

Costa Rica and Chiapas were chosen as the sites to implement the study because both sites use paid health promoters (primary health care workers) within the official systems, and have an already existing infrastructure and programs for patients with type 2 diabetes and arterial hypertension. Other countries in Mesoamerica have limited or variable infrastructure and programs for chronic disease care and prevention as their health care systems are primarily oriented toward maternal and child health and infectious disease. The intervention and comparison health centers in each setting were selected for inclusion in the study in coordination with health authorities, aiming to be similar with respect to the services offered (laboratory, pharmacy and clinical care), number of patients served, and socio-economic status of the population. In each country setting, intervention and comparison group health centers were in different parts of the city in order to avoid contamination. The health center staff provided the research study team with a list of patients identified as having diabetes, hypertension or both conditions; the research team was in charge of selecting eligible patients at both the intervention and comparison health centers who fit the study’s inclusion criteria. The process for selecting patient participants was done similarly in both countries.

In Costa Rica, participants were recruited at health centers of the *Caja Costarricense de Seguridad Social*, the Costa Rican Social Security Institute, that offers insurance coverage to the whole population. In Tuxtla Gutiérrez, Chiapas participants were recruited at health centers of the Secretary of Health, operating under *Seguro Popular*, or popular insurance, that covers about a quarter of the population in Tuxtla Gutiérrez. In addition in Mexico, a cash transfer program called *Oportunidades* has been operating for over a decade in which patients have several requirements, including attending health promotion sessions on different topics. Documented differences in the health care context and resources between the countries included: human resources, medications, and routine care provided to patients with chronic conditions.

The study protocol was reviewed and approved by the Institutional Review Boards of the RAND Corporation (study # 2010–0700), the Institute of Nutrition of Central America and Panamá, the University of Costa Rica, the Costa Rican Social Security Institute and the Health Institute of the State of Chiapas.

### Study participants

Sample size was calculated at 75 patients in each of the intervention and comparison groups in each country, and the study teams aimed to recruit up to 90 patients to account for drop-outs and loss to follow-up. The sample size was calculated based on the experience of the pilot study conducted in Guatemala in which 75 patients were recruited for a study with a similar time frame and patients experienced significant reduction in mean systolic blood pressure of 27.2 mmHg.

Individual participants were selected from patients registered at public health centers who had a diagnosis of hypertension and/or type 2 diabetes, certified by a physician. The selection of patients to participate in the study was consecutive. All potential participants were offered information about the research and those who were interested granted written consent to participate.

The inclusion criteria for participating in the study were: being 21 years or older, residing in the area served by the selected health center, literate, and diagnosed with hypertension and/or type 2 diabetes. Intervention group participants also had to be willing to comply with proposed educational activities, including attendance at training sessions with support staff based at the health center.

The exclusion criteria were: previously experienced complications from diabetes (chronic kidney disease, peripheral circulation of the retina, organ damage), a history of stroke (thrombosis, ischemia, aneurysm) or cardiac problems (angina, infarction). Other criteria for exclusion included the presence of disease or mental impairment preventing their understanding of the instructions provided as part of the educational strategy; physical defect, pregnancy, or disability preventing regular physical exercise. A screening questionnaire was used with patients to define who should be excluded from the study and patient records were then used to verify the information.

Patients in the usual care group received a clinic visit with their primary care physician and laboratory tests. In Costa Rica, the usual care for patients with diabetes is every 3 months and for hypertension is every 4 months and patients receive their medications the day of their clinic visit. For Chiapas, patients with diabetes and hypertension were seen every month and at the same monthly visit they have relevant lab tests done and pick up their medications.

### Outcome measures

The outcomes measured in this study are presented in Fig. 1. The cognitive and behavioral measures were knowledge, self-efficacy, stage-of-change, dietary behavior and physical activity. A knowledge questionnaire was designed by project staff based on the key concepts presented in the group education sessions and was used at baseline and 8-months focusing on the topics covered in the health education intervention (maximum of 45 points). A sample question from the knowledge questionnaire in the topic of diabetes was to mark off which of the following ways can let us know that foods are high in carbohydrates – food that: a) are very watery, b) are sweet tasting and do not say “without sugar”, c) are doughy, pasty or floury, and d) have a lot of fiber. A scale of self-efficacy (with questions relevant for patients with hypertension and/or diabetes) was created based on an instrument titled Self-Efficacy for Diabetes designed for diabetics at the Stanford Patient Education Reference Center [20], with a maximum of 15 points. A sample question from the self-efficacy scale was: how confident do you feel that you can control your condition so that it does not interfere with the things you want to do? A stages-of-change scale was designed using 8 questions focused on topics emphasized in the group education sessions (medication adherence and care, diet, physical activity, family communication, and stress management); the scale ranged from 0–32 points. A sample question on stages-of-change was to mark off the stage in which the patient finds his or herself for: taking medications at the recommended time and in the recommended amount. A diet behavior index was created using a food consumption and dietary behavior questionnaire designed by the project team, with a maximum of 14 points. A sample question from the diet questionnaire was: do you add salt to your food after it has been prepared (never, always, sometimes?). Physical activity was captured using the International Physical Activity Questionnaire (IPAQ) Short Form (www.ipaq.ki.se) and was summarized as meeting the recommended minutes of aerobic activity per week: at least 150 min of moderate-level activity or 75 min of vigorous-level activity [21]. Instruments developed by staff for the intervention were validated for understandability with 5–10 patients in each country setting; the patients attended the selected health centers but were not study participants.

The clinical outcomes were: body mass index, systolic blood pressure (mmHg), diastolic blood pressure (mmHg), and fasting blood glucose. All clinical outcomes were captured while fasting. Body mass index was calculated as (kg/m^2^). Resting blood pressure was measured after a 10 min rest in the seated position with a calibrated digital monitor (CITIZEN, Model CH 432) and each country team used its own monitor for the study; the average of three measurements taken 2 min apart, within 6 mmHg, was used. Plasma glucose was measured in the primary health care laboratories using conventional methods in Costa Rica, while in Mexico, the finger-prick method was used.

### Individual patient characteristics

Individual patient characteristics that were included as co-variates in the regression analyses were: age (in years), sex, formal education level (years studied), works currently or is not currently employed, lives alone or not, distance from the health center (categorized as being less than 1 km, 1–2, 2–4 or greater than 4 km to the primary care health center), if diabetic or not, and if hypertensive or not.

### Analysis

The database was constructed using REDCap, [22] and data were double-entered on-site in Costa Rica and in Chiapas. Data were compared for inconsistencies and corrected and then were transferred to Stata SE version 12 (StataCorp LP, College Station, TX, USA) for statistical analysis.

The analysis was conducted separately for the two sites given the substantial difference in contexts and patient populations. Descriptive statistics were used to compare intervention and comparison group participants at baseline; an unpaired t-test with equal variances was used to measure differences between the two groups for continuous variables, and a *X*^2^ analysis was used for categorical variables.

For the impact evaluation, the generalized estimating equation (GEE) method, with robust standard errors, was used comparing baseline to end-point, adjusting for baseline. The treatment and comparison groups were coded with a (1/0) variable, and an interaction with time was generated. An intention-to-treat analysis was conducted. For the regression analyses, a priori models were defined and all covariates included simultaneously. Linear regression models were used for: body mass index, systolic blood pressure, diastolic blood pressure, blood glucose, knowledge, self-efficacy, stage-of-change, and diet index. A logistic regression model was used for whether recommended physical activity guidelines were met or not. For the intervention group only, a multivariate regression analysis was conducted to test for a dose–response relationship of exposure to educational sessions and health outcomes, comparing changes for outcomes at 8-month follow-up to baseline; for the dose–response analysis, linear regression models were used for all outcomes.

## Results

The two study sites presented differences in health center infrastructure and routine care for patients with hypertension and/or diabetes. In both countries, national treatment guidelines include risk classification by condition; in Costa Rica, patients’ appointments were scheduled based on risk classification whereas in Chiapas, patients’ appointments were scheduled on a monthly basis as they were linked to 30-day prescription refills. Both countries had a basic list of medications in the health centers to treat hypertension, which included calcium blockers, beta-receptor blockers, angiotensin converting enzyme inhibitors, and in Costa Rica, diuretics and angiotensin receptor blockers. For diabetes, oral hypoglycemic drugs and insulin were available in the health centers.

Table 2 presents baseline patient characteristics for the intervention and comparison groups in both Costa Rica and Chiapas. Comparing intervention and comparison groups in Costa Rica, intervention group participants were: more likely to live a short distance from a primary care health center (*p* < 0.001) and more likely to be normal weight (*p* = .012). Comparing intervention and comparison groups in Chiapas, intervention group participants were: significantly younger (51.6 vs. 56.7 years; *p* = .006), more likely to be female (92 vs. 78 %; *p* = .01), more likely to live at a greater distance from a primary care health center (*p* = 0.004), more likely to be overweight or obese (*p* = .001), and less likely to have diabetes (*p* < .001) and both conditions (diabetes and hypertension) (*p* < .001) than the comparison group. On average, participants in Costa Rica were older (63 vs. 53 years), had more years of formal education (9 vs. 4.5 years), and were more likely to have high or medium global cardiovascular disease risk (46 vs. 24 %) than those in Chiapas.

**Table 2:** Baseline Socio-Demographic Characteristics of Patients Enrolled in the Primary Health and Community Support Model to Lower the Risk of CVD in Tuxtla Gutiérrez, Chiapas, Mexico and San José, Costa Rica

Variable	Costa Rica		*p*-value	Chiapas		*p*-value
	Intervention Group	Comparison Group		Intervention Group	Comparison Group	
N	84	86		87	81	
Age in years (SD)	64.0 (8.9)	62. 6 (9.6)	.32	51.6 (11.5)	56.7 (12.2)	.006
Female (%)	59.5 %	68.6 %	.22	92.0 %	77.8 %	.01
Education, years studied	9.74	8.59	.09	4.78	4.38	.55
Works (%)	16.3 %	9.4 %	.19	29.9 %	32.1 %	.76
Lives Alone (%)	9.6 %	9.4 %	.96	10.3 %	3.7 %	.10
Distance from health center (%)			<.001			0.004
*Less than 1 km*	39.3 %	10.6 %		23.0 %	28.4 %	
* 1-2 km*	50.0 %	45.9 %		14.9 %	34.6 %	
* 2-4 km*	7.1 %	37.7 %		29.9 %	22.2 %	
* >4 km*	3.6 %	5.9 %		32.2 %	14.8 %	
Global Cardiovascular Risk Level (%)			.16			.24
* Low*	49.3 %	57.9 %		81.7 %	70.4 %	
* Medium*	32.5 %	34.2 %		11.0 %	17.3 %	
* High*	18.2 %	7.9 %		7.3 %	12.3 %	
Diabetes (%)	60.7 %	73.3 %	.08	44.8 %	80.3 %	<.001
Hypertension (%)	89.3 %	81.4 %	.15	64.4 %	50.6 %	.07
Both (%)	51.2 %	54.7 %	.65	9.2 %	30.9 %	<.001

Table 3 presents baseline measures of outcome variables of interest comparing intervention and comparison groups in both Costa Rica and Chiapas. On average, participants in Costa Rica had substantially lower fasting glucose measures but were otherwise comparable. Comparing intervention and comparison groups at baseline in Costa Rica, intervention group participants were: significantly more likely to have high systolic (*p* < 0.001) and diastolic (*p* = 0.001) blood pressure, and to meet the recommendations for weekly minutes of physical activity (*p* = 0.03). Comparing intervention and comparison groups at baseline in Chiapas, intervention group participants were: significantly more likely to get the recommended amount of physical activity (*p* < 0.001), significantly more likely to report healthy dietary behavior (*p* = 0.01), and significantly more likely to have a higher composite knowledge score (*p* = 0.04) than the comparison group.

**Table 3:** Baseline Measures of Outcome Variables for Study Participants in Chiapas and Costa Rica

		Costa Rica Intervention Group		Costa Rica Comparison Group			Chiapas Intervention Group		Chiapas Comparison Group	
Variable	N	Mean (95 % CI)	N	Mean (95 % CI)	*p*-value	N	Mean (95 % CI)	N	Mean (95 % CI)	*p*-value
Cognitive and Behavioral Outcomes										
Knowledge Measure (0–45 points)	80	32.9 (31.7, 34.1)	86	32.9 (31.9, 33.9)	0.99	71	29.3 (27.7, 30.9)	80	27.4 (26.2, 28.5)	0.04
Self-efficacy Measure (0–15)	83	12.9 (12.4, 13.5)	86	12.6 (12.1, 13.1)	0.33	87	13.4 (13.0, 13.8)	81	13.1 (12.6, 13.6)	0.47
Stages of Change Measure (0–32)	76	22.6 (21.6, 23.7)	83	23.8 (22.7, 25.0)	0.14	86	22.3 (21.1, 23.4)	81	23.1 (22.1, 24.1)	0.27
Diet Index (0–14)	75	10.7 (10.2, 11.2)	81	10.9 (10.4, 11.4)	0.56	86	11.5 (11.1, 11.9)	81	10.5 (10.2, 10.9)	0.01
Meets Recommended minutes of physical activity per week (%)	80	66.25 %	86	50 %	0.03	87	75.9 %	81	45.7 %	<0.001
Clinical Outcomes										
Body mass index (kg/m^2)^ (%)	74		80		.012	87		81		.001
Normal weight (18.5 < 25)		17.6 %		10 %			8.4 %		28.4 %	
Overweight (25 < 30)		50 %		33.8 %			41.0 %		44.4 %	
Obese (> = 30)		32.4 %		56.2 %			50.6 %		27.2 %	
Systolic BP (mmHg)	76	140.4 (135.8, 144.9)	76	127.6 (124.1, 131.2)	<.001	82	134.2 (128.9, 139.4)	81	136.0 (130.8, 141.2)	0.61
Diastolic BP (mmHg)	76	78.8 (70.3, 74.7)	76	72.5 (76.5, 81.1)	.001	82	75.7 (73.3, 78.1)	81	76.0 (73.6, 78.4)	0.84
Fasting Blood Glucose	70	127.4 (114.7, 140.1)	76	122.4 (113.6, 131.2)	0.52	83	145.8 (130.8, 160.8)	80	155.0 (139.4, 170.5)	0.40

At baseline, in Costa Rica 84 participants were in the intervention group and 86 were in the comparison group, and in Chiapas 87 were in the intervention group and 81 were in the comparison group. The two sites presented differences in participation in the healthy lifestyle intervention. The average number of healthy lifestyle group education sessions attended in Costa Rica was 1.8 (SD: 2.0) and in Chiapas was 4 (SD: 2.2). In Costa Rica, 7 % of participants in the intervention group attended all 6 sessions whereas in Chiapas the percentage was 44 %. In Costa Rica 42 % of patients enrolled in the intervention group did not attend any session and in Chiapas that percentage was 13 %. The primary reasons reported for having difficulty attending the group education sessions were: health problems, other commitments including taking care of family members or work, weather, medical appointments, and difficulties in communication with study participants because of changes in contact information. At 8-month follow-up, in Costa Rica 47 participants from the intervention group and 59 from the comparison group presented to the health center for final assessment; and in Chiapas, at 8-months outcomes were captured for 58 participants from the intervention group and 80 from the comparison group.

Table 4 presents impact evaluation results comparing intervention and comparison groups at the end of the healthy lifestyle intervention, adjusting for age, sex, diabetic, hypertensive, years of formal schooling, distance to the health center, employed, and lives alone. In Costa Rica, the intervention group showed significant reduction in systolic blood pressure (Coefficient = −8.26, *p* = 0.003) and diastolic blood pressure (Coefficient = −5.66, *p* < 0.001), and borderline significant reduction in fasting glucose (Coefficient = −9.34, *p* = 0.05) relative to the comparison group at the end of the 8-month intervention. The intervention group also showed significant improvement in the stages-of-change measure as compared to the comparison group at end-point (Coefficient = 3.37, *p* < .001). No significant change was observed for self-efficacy, physical activity, diet index, knowledge, or body mass index in intervention vs. comparison group participants.

**Table 4:** Assessment of Cognitive, Behavioral, and Clinical Impact of the Healthy Lifestyle Group Education Intervention in Costa Rica and Chiapas

	Costa Rica		Chiapas	
	Coefficient^a^	*p*-value	Coefficient^a^	*p*-value
Cognitive and Behavioral Outcomes				
Knowledge	1.24	0.17	2.38	0.06
Self-efficacy	−0.15	0.69	0.22	0.51
Stages of change	3.37	<.001	6.15	<.001
Diet index	0.63	0.07	0.54	0.1
Physical activity	−0.87	0.28	-.89	0.26
Clinical Outcomes				
Body mass index	0.37	0.32	−0.01	0.98
Systolic BP	−8.26	0.003	3.48	0.30
Diastolic BP	−5.66	<.001	−0.78	0.68
Glucose	−9.34	0.05	−13.3	0.21

^a^Adjusted for age, sex, years of formal schooling, distance to the health center, working, lives alone and diabetic and/or hypertensive disease status, and baseline values

In Chiapas, the intervention group showed significant improvement in the stages-of-change measure as compared to the comparison group at the end of the intervention (Coefficient = 6.15, *p* < .001). No significant change was observed for self-efficacy, physical activity, diet index, composite knowledge score, or clinical outcomes in intervention vs. comparison group participants.

A dose–response analysis was conducted for intervention group participants in each of the two sites. In Chiapas, for those participants attending more healthy lifestyle education sessions, a significantly greater reduction in systolic (Coefficient: −9.14; *p* < 0.001) and diastolic (Coefficient: −3.72; *p* = 0.002) blood pressure was observed.

For all other outcomes, no dose–response relationship was observed.

## Discussion

The main strength of this study is that it is one of few studies conducted in primary care health centers in Mesoamerica aimed at reducing cardiovascular disease risk for patients with type 2 diabetes and arterial hypertension. Likewise, this is one of the few studies in the region focused on healthy lifestyle promotion that includes participants with more than one chronic condition.

We found significant improvements in the stage-of-change in which intervention participants considered themselves to be in both countries. In Costa Rica, participants in the intervention group improved on average 3 points more on the stage-of-change scale than those in the comparison group, adjusting for baseline values and co-variates. In Chiapas, participants in the intervention group improved on average 6 points more than those in the comparison group on the stage-of-change scale. In Costa Rica, being in the intervention group was associated with a significant reduction in systolic and diastolic blood pressure and borderline significant improvement in glucose control. On average, systolic blood pressure was reduced 8 mmHg and diastolic blood pressure was reduced 5 mmHg more for the intervention vs. the comparison group from the start to the end of the intervention, adjusting for baseline values and co-variates. In a dose–response analysis, the number of educational sessions attended by participants in Chiapas was associated with a significant reduction in systolic and diastolic blood pressure at 8-months. There was no significant improvement in physical activity, dietary practices, self-efficacy, or body mass index reduction. Aside from the initial stage-of-change improvement observed in the intervention group in both settings, the observed outcomes did not follow what was expected according to the conceptual model of improved stage-of-change, self-efficacy, and knowledge leading to behavior change and improved clinical outcomes.

The notable differences in the patient populations and the health infrastructure and context in the two settings appear to have had a major influence on the outcomes of the intervention. Part of the reason why the intervention showed the effects in clinical outcomes in Costa Rica but not in Mexico is visible from patterns in Table 2; the people in the intervention group in Costa Rica were older and with higher baseline blood pressure than those in Chiapas and therefore had more potential for improvement. In addition, the available health resources in Costa Rica and Chiapas are different and patients with increased knowledge, self-efficacy, and stage activation in each country may have faced differences in accessing medications.

In Chiapas, dropout during the course of the intervention was lower than for Costa Rica and overall attendance in the group education sessions was higher throughout the intervention as compared to Costa Rica. The lower attendance in Costa Rica may be related to the fact that the participants in the study had lived with the condition for a longer time period than those in Chiapas who were more recently diagnosed and had previously had fewer opportunities to attend educational sessions about their condition. Higher attendance in Chiapas may have been due in part to the fact that participants in this study had coverage through the national program called *Oportunidades* which encourages participation in health education, as it is a factor considered in its provision of cash transfers; while participation in this study was explicitly not linked to cash transfers, patients in Chiapas may have been accustomed to regular participation in health education sessions. In addition, in Costa Rica, the health care teams routinely provide information to patients with chronic conditions – typically on an individual basis - and the participants in this study may have decided that they were not going to obtain anything more from attending the group education sessions delivered in this study, even though the methodology of the small groups sessions in this study was different from that of other group sessions. In Chiapas, on the other hand, patients may have had higher participation due to being more accustomed to participating in activities organized by the health center, including talks and campaigns on specific health priorities. The 30-day medication refill in Chiapas may have also had an influence on attendance.

The study had a number of limitations. We noted substantial differences in the health care contexts and patient populations both within and between the two settings. We conducted the analysis separately in order to address the differences between the two countries, however, for intervention and comparison groups in the same country, we adjusted for available socio-demographic variables in regression analyses. It is possible that we did not fully adjust for the differences in patient characteristics as we were limited to the socio-demographic variables captured at baseline. In the future, it would be preferable to enroll patients with more similar characteristics in the different country settings – and in both the intervention and comparison groups - to measure more precisely the effect of the intervention. For example, in Chiapas, the patients on average had fewer years of education and a higher proportion of people working than in Costa Rica. Another factor that we did not capture in this analysis is the different experiences that patients received in their usual care, as we did not track number of clinic visits, medications, and assessment and follow-up at clinic visits. And in comparing the two country experiences, it should be noted that while the brand and model of equipment of the blood pressure monitor was the same, the instrument itself was different.

A key limitation is that a large number of participants in both countries did not continue through to the end of the intervention, and this was especially marked in Costa Rica. In Costa Rica the loss to follow up was sizeable in both arms whereas in Chiapas, at 8-months, only one patient was lost in the comparison group and one third were lost in the intervention group. Understanding reasons that participants did not continue through to the end of the intervention is an important topic that warrants future research. In addition, the sample size for the study had been calculated based on the intervention previously implemented in Guatemala in which the population had different characteristics; as such, the study was underpowered to detect significant change in the primary clinical outcomes.

Another limitation is that this study did not capture measures related to participants’ access to resources that would enable them to make changes in their diet and physical activity; we propose including these variables in future studies, using variables such as those proposed in the Capability Approach [23]. In addition, due to the non-significant findings in health behavior, we propose that future interventions build on the findings of patients’ improvement with respect to activation, as shown by the stage-of-change variable, and that there be a concerted effort to understand barriers that patients face in converting their intent to change into actual change in diet and physical activity.

The study served both as a test of an intervention aimed at addressing a considerable public health problem and also provided an opportunity to identify factors that may be important for future healthy lifestyle interventions. For example, in both sites, enrollment by men - as compared to women – was lower. This finding spurred a new study that was conducted from 2013–14 to understand barriers to men’s participation and the opportunity to include family members in the study to increase its reach [24].

## Conclusion

To conclude, the study demonstrates that group education interventions at health centers have the potential to improve the stage in which the patient considers himself or herself to be with respect to their capacity to manage their condition, and may also improve knowledge and clinical outcomes. In the future, it will be essential to dedicate time and resources to understand the best ways to reach a representative group of the patient population affected by diabetes and hypertension. Also, given the large number of participants who did not continue to the end of the study in both sites, and the low number of sessions attended in Costa Rica, it will be important to consider adaptations to the intervention that will allow for patients to be given the opportunity to learn the content from all of the education sessions, if group education sessions are not their preferred medium.

## Abbreviations

CVD: Cardiovascular disease
INCAP: Institute of Nutrition of Central America and Panamá
NHLBI: National Heart, Lung and Blood Institute
